# One-fits-all pretreatment protocol facilitating Fluorescence In Situ Hybridization on formalin-fixed paraffin-embedded, fresh frozen and cytological slides

**DOI:** 10.1186/s13039-019-0442-4

**Published:** 2019-06-17

**Authors:** Shivanand O. Richardson, Manon M. H. Huibers, Roel A. de Weger, Wendy W. J. de Leng, John W. J. Hinrichs, Ruud W. J. Meijers, Stefan M. Willems, Ton L. M. G. Peeters

**Affiliations:** 1 Department of Pathology, University Medical Center Utrecht, Heidelberglaan 100, 3584 CX Utrecht, The Netherlands; 2 Department of Genetics, University Medical Center Utrecht, Utrecht, The Netherlands; 3 Department of Pathology, Erasmus Medical Center Rotterdam, Rotterdam, The Netherlands

**Keywords:** Fluorescence In Situ Hybridization, FISH, Pretreatment, Formalin-fixed paraffin-embedded, Fresh frozen, Cytological, Molecular pathology

## Abstract

**Background:**

The Fluorescence In Situ Hybridization (FISH) technique is a very useful tool for diagnostic and prognostic purposes in molecular pathology. However, clinical testing on patient tissue is challenging due to variables of tissue processing that can influence the quality of the results. This emphasizes the necessity of a standardized FISH protocol with a high hybridization efficiency. We present a pretreatment protocol that is easy, reproducible, cost-effective, and facilitates FISH on all types of patient material simultaneously with good quality results.

During validation, FISH analysis was performed simultaneously on formalin-fixed paraffin-embedded, fresh frozen and cytological patient material in combination with commercial probes using our optimized one-fits-all pretreatment protocol. An optimally processed sample is characterized by strong specific signals, intact nuclear membranes, non-disturbing autofluorescence and a homogeneous DAPI staining.

**Results:**

In our retrospective cohort of 3881 patient samples, overall 93% of the FISH samples displayed good quality results leading to a patient diagnosis. All FISH were assessed on quality aspects such as adequacy and consistency of signal strength (brightness), lack of background and / or cross-hybridization signals, and additionally the presence of appropriate control signals were evaluated to assure probe accuracy. In our analysis 38 different FISH probes from 3 commercial manufacturers were used (Cytocell, Vysis and ZytoLight). The majority of the patients in this cohort displayed good signal quality and barely non-specific background fluorescence on all tissue types independent of which commercial probe was used.

**Conclusion:**

The optimized one-fits-all FISH method is robust, reliable and reproducible to deliver an accurate result for patient diagnostics in a lean workflow and cost-effective manner. This protocol can be used for widespread application in cancer and non-cancer diagnostics and research.

## Background

**Fig. 1:**
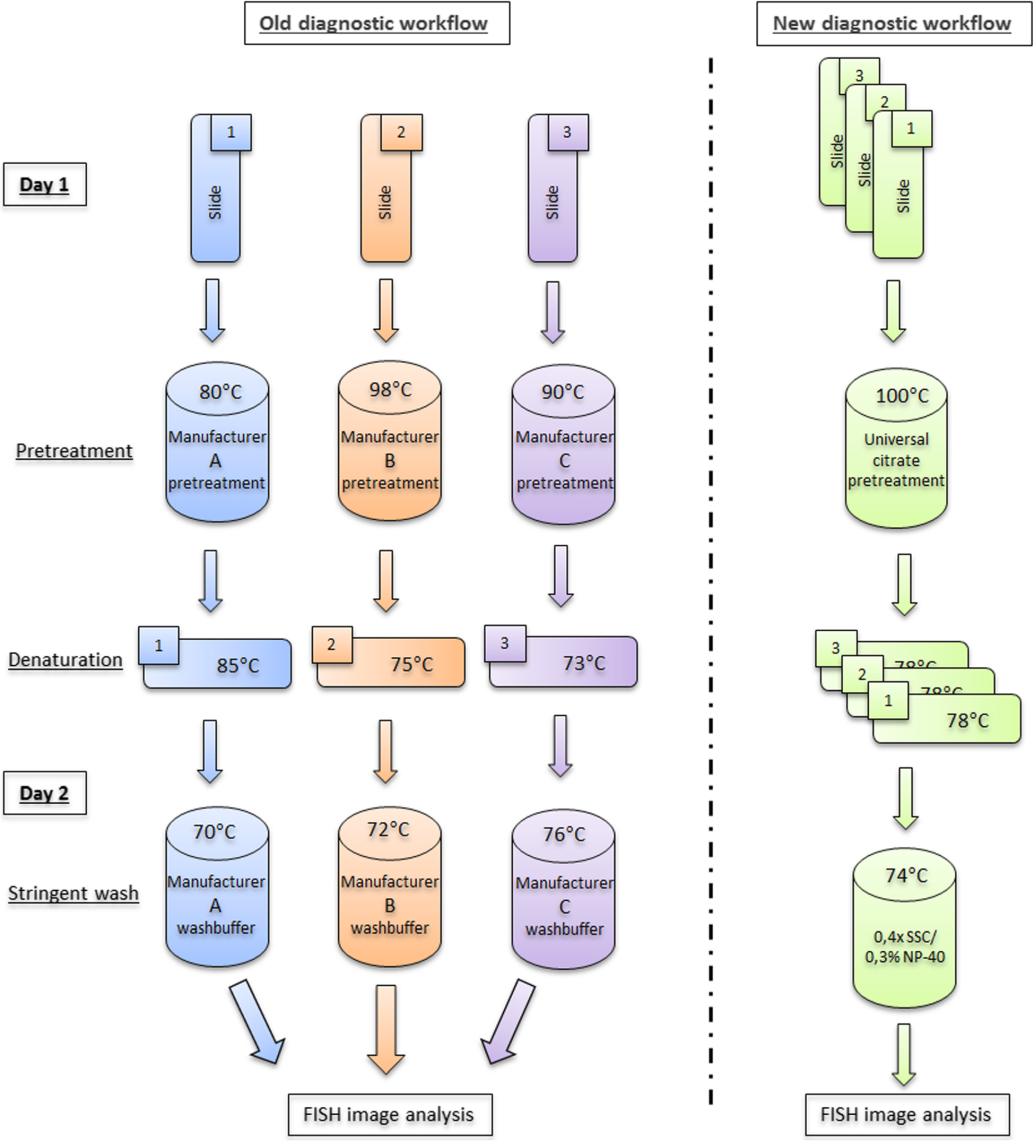
A comparison of the FISH diagnostic workflows. The old diagnostic workflow comprised of various commercial methods from different manufacturers that would be used simultaneously depending on the patient requests. The new diagnostic workflow uses one in-house developed protocol for all patient samples. Additionally all reagents excluding probes are prepared in-house, making it very cost effective

Fluorescence In Situ Hybridization (FISH) is a widely used approach to localize the presence or absence of a specific genetic aberration that may potentially be associated with tumor types or subtypes, cellular morphology, disease prognosis, or response to targeted therapy [1]. FISH is a molecular cytogenetic method that has advantages over metaphase chromosome analysis by karyotyping because it can be applied on both dividing (metaphase) and non-dividing cells (interphase), the resolution is better whereby detection of a small genetic aberration can be achieved and it has a lower threshold for detecting small populations of abnormal cells (in low tumor percentage or low mosaicism samples) [2]. By using fluorescent DNA probes to hybridize entire chromosome regions or single unique sequences, it serves as a powerful adjunct to classic molecular cytogenetics and pathology diagnostics [3]. FISH involves the binding (or annealing) of fluorescence labeled, target-specific nucleic acid probes to their complementary DNA sequences and the subsequent visualization of these probes within cells in the tissue of interest [4]. The detection of chromosomal rearrangements, amplifications and deletions by FISH is well-accepted as a robust and reliable technique [5]. The pretreatment procedure contains essential steps to obtain an optimal fluorescent signal which makes it crucial when conducting FISH analysis. The technical challenge of applying FISH lies within the pretreatment protocol. An efficient pretreatment protocol should expose the target genes and allow the penetration of the probes without significantly altering the integrity and morphology of the tissue [6]. In the current study we have developed a general pretreatment method in order to standardize our FISH procedure for many tissue types and probes produced by different manufacturers (Fig. 1). The aim of this methods paper is to describe and introduce our robust, in-house developed and cost-effective general pretreatment FISH procedure that is validated according to the diagnostics quality criteria within the molecular pathology diagnostics and research.

## Results

### Sample characteristics

Of all 3881 patient samples included 3376 samples (87%) were formalin-fixed paraffin-embedded (FFPE) material, 466 samples (12%) were cytological specimen and 39 samples (1%) were fresh frozen (FF).

### FISH analyses results

Of the full cohort, 87% of the samples were tested with Cytocell probes, 10% with ZytoLight probes and 1% with Vysis probes (Table 1).

**Table 1:** FISH results of 3881 FFPE, FF and cytological patient samples tested with the optimized pretreatment protocol in combination with Vysis, Cytocell and ZytoLight commercial probes

Gene of Interest (Chromosomal location)	Probe	Manufacturer	Diagnostic requests (# slides)	Cut off criteria for aberrant signal pattern	# slides with interpretable results (%)	# slides with uninterpretable results (%)
ALK (2p23.2-p23.1)	ALK Breakapart	Cytocell	219	15%	195 (89%)	24 (11%)
BCL2 (18q21.33-q22.1)	BCL2 Breakapart	Cytocell	312	10%	300 (96%)	12 (4%)
BCL6 (3q27.3-q28)	BCL6 Breakapart	Cytocell	269	10%	261 (97%)	8 (3%)
CEP6 (6p11.1-q11)	CEP 6 (D6Z1)	Vysis	30	< 2copies	30 (100%)	–
CHOP (DDIT3) (12q13.3)	CHOP (DDIT3) Breakapart	Cytocell	18	10%	16 (89%)	2 (11%)
DDIT3 (12q13.3-q14.1)	SPEC DDIT3 Dual Color Break Apart Probe	ZytoLight	17	10%	17 (100%)	–
c-MET (MET) (7q31.2)	c-MET (MET) Amplification	Cytocell	94	*	92 (98%)	2 (2%)
cMYC (MYC) (8q24.21)	cMYC (MYC) Breakapart	Cytocell	346	10%	336 (97%)	10 (3%)
IGH (14q32.33) / cMYC (MYC) (8q24.21)	IGH/cMYC (MYC) Translocation, Dual Fusion	Cytocell	315	10%	302 (96%)	13 (4%)
COL1A1 (17q21.33) / PDGFB (22q13.1)	SPEC COL1A1/PDGFB Dual Color Dual Fusion Probe	ZytoLight	12	10%	12 (100%)	–
CCND1 (11q13.3)	CCND1 Breakapart	Cytocell	42	10%	40 (95%)	2 (5%)
CCND1 (11q13.3) / CEP11 (11p11.11-q11)	LSI Cyclin D1 (11q13) Spectrum Orange/ CEP 11 Spectrum Green	Vysis	9	10%	9 (100%)	–
CCND1 (11q13.2-q13.3)	SPEC CCND1 Dual Color Break Apart Probe	ZytoLight	9	10%	9 (100%)	–
ETV6 (12p13.2)	ETV6 Break Apart FISH Probe	Vysis	33	10%	31 (94%)	2 (6%)
EWSR1 (22q12.1-q12.2)	EWSR1 Breakapart	Cytocell	47	10%	42 (89%)	5 (11%)
EWSR1 (22q12.1-q12.2)	SPEC EWSR1 Dual Color Break Apart Probe	ZytoLight	46	10%	43 (93%)	3 (7%)
EWSR1 (22q12.1-q12.2) / FLI1 (11q24.3)	SPEC EWSR1/FLI1 TriCheck Probe	ZytoLight	20	10%	20 (100%)	–
FOXO1 (13q14.1)	FOXO1 Break Apart FISH Probe	Vysis	30	10%	30 (100%)	–
FUS (16p11.2)	FUS Breakapart Probe	Cytocell	24	10%	24 (100%)	–
FUS (16p11.2)	SPEC FUS Dual Color Break Apart Probe	ZytoLight	24	10%	21 (87%)	3 (13%)
HER2 (ERBB2) (17q12)	HER2 (ERBB2) Amplification	Cytocell	150	**	149 (99%)	1 (1%)
MALT1 (18q21.31-q21.32)	MALT1 Breakapart	Cytocell	25	10%	25 (100%)	–
MALT1 (18q21.31-q21.32)	SPEC MALT1 Dual Color Break Apart Probe	ZytoLight	25	10%	25 (100%)	–
MAML2 (11q21)	SPEC MAML2 Dual Color Break Apart Probe	ZytoLight	33	10%	32 (97%)	1 (3%)
MDM2 (12q15)	MDM2 Amplification	Cytocell	27	***	26 (96%)	1 (4%)
IRF4,DUSP22 (6p25.3)	SPEC IRF4,DUSP22 Dual Color Break Apart Probe	ZytoLight	17	10%	17 (100%)	–
MYB (6q23.2-q23.3)	SPEC MYB Dual Color Break Apart Probe	ZytoLight	83	10%	79 (95%)	4 (5%)
N-MYC (2p24.3), LAF4(AFF3) (2q11.2)	N-MYC (MYCN) Amplification	Cytocell	133	****	133 (100%)	–
PLAG1 (8q12.1) / CTNNB1 (3p22.1)	PLAG1/CTNNB1 Fusion	Cytocell	79	10%	75 (95%)	4 (5%)
CEP X (Xp11.1 - q11.1), CEP Y (Yp11.1 - q11.1), CEP 18 (18p11.1 - q11.1)	Prenatal X, Y and 18 Enumeration Probe	Cytocell	39	≥3 copies	39 (100%)	–
13 unique sequence (13q14.2), CEP 18 (18p11.1 - q11.1), 21 unique sequence (21q22.13)	Prenatal 13, 18 and 21 Enumeration Probe	Cytocell	59	≥3 copies	58 (98%)	1 (2%)
RET (10q11.21)	RET Breakapart	Cytocell	575	15%	500 (87%)	75 (13%)
ROS1 (6q22.1)	ROS1 Plus Breakapart	Cytocell	555	15%	461 (83%)	94 (17%)
SYT (SS18) (18q11.2)	SYT (SS18) Breakapart	Cytocell	35	10%	28 (80%)	7 (20%)
SS18 (18q11.2)	SPEC SS18 Dual Color Break Apart Probe	ZytoLight	35	10%	35 (100%)	–
TFE3 (Xp11.23)	SPEC TFE3 Dual Color Break Apart Probe	ZytoLight	15	10%	15 (100%)	–
USP6 (17p13.2)	SPEC USP6 Dual Color Break Apart Probe	ZytoLight	59	10%	55 (94%)	4 (6%)
YWHAE (17p13.3)	SPEC YWHAE Dual Color Break Apart Probe	ZytoLight	21	10%	21 (100%)	–
Total	38 probes	3 manufacturers	3881		3603 (93%)	278 (7%)

*c-MET according to Garcia L. University of Colorado defined criteria. Dual-probe MET/CEP7 ratio < 1.8 is not amplified, ratio ≥ 1.8 - ≤2.2 is low level MET amplification, ratio > 2.2 - ≤5.0 is intermediate level MET amplification, ratio ≥ 5.0 is high level MET amplification [7]. ** HER2 according to ASCO–CAP Guidelines. Her2 is amplified when; Single-probe average HER2 copy number ≥ 6.0 signals/cell, Dual-probe HER2/CEP17 ratio ≥ 2.0 with an average HER2 copy number ≥ 4.0 signals/cell, ratio ≥ 2.0 with an average HER2 copy number < 4.0 signals/cell, ratio < 2.0 with an average HER2 copy number ≥ 6.0 signals/cell. Her2 is equivocal when; Single-probe ISH average HER2 copy number ≥ 4.0 and < 6.0 signals/cell, Dual-probe HER2/CEP17 ratio < 2.0 with an average HER2 copy number ≥ 4.0 and < 6.0 signals/cell. Her2 is negative when; Single-probe average HER2 copy number < 4.0 signals/cell, Dual-probe HER2/CEP17 ratio < 2.0 with an average HER2 copy number < 4.0 signals/cell [8]. *** MDM2/CEP12 ratio ≥ 2.0 is considered amplified, < 2.0 not amplified, and cases displaying > 2 signals of both probes and an MDM2 ratio < 2.0 polysomic for chromosome 12 [9]. ****MYCN-Status according to the DCOG NBL 2009 treatment protocol. Dual-probe MYCN/CEP2 ratio 1 is not amplified, MYCN/CEP2 ratio 1.5–4 indicates a gain, MYCN/CEP2 ratio > 4 is amplified (DCOG NBL 2009 Final Version Amendment 1)

During the verification phase, all new probes were initially assessed on 10 normal healthy tissue samples to determine the adequacy and consistency of signal strength (brightness), lack of background and / or cross-hybridization signals, presence of appropriate control signals, sensitivity, specificity, reproducibility and stability between runs from which it was observed that < 10% (range 1–7%) of normal healthy tissue cells displayed an aberrant signal pattern (break apart, deletion, fusion, or amplification), due to biological heterogeneity or technical artefacts. However the threshold of 10% aberration in normal tissue was never exceeded. Only those probes with interpretable results in ≥95% of the cases were implemented in the routine diagnostics. In addition the probes were also tested on tumor tissue with a known or suspected for the genetic aberrations to assess the accuracy of diagnosis.

### Image robustness for diagnostic analysis

Overall in our diagnostic cohort, 3609 slides (93%) of the tested patient samples displayed good quality results leading to a patient diagnosis, whereas 272 slides (7%) displayed uninterpretable results (Table 1). All diagnostic FISH requests were assessed on quality aspects such as adequacy and consistency of signal strength (brightness), lack of background and / or cross-hybridization signals, and additionally the presence of appropriate control signals to assure probe accuracy. Patients with interpretable results are considered for further treatment or diagnosis whereas uninterpretable results are not. Uninterpretable results are characterized by a poor cellular morphology, loss of /or low fluorescent signal quality, and /or high background fluorescence signals. In the case of uninterpretable results alternative diagnostic experiments are conducted.

**Fig. 2:**
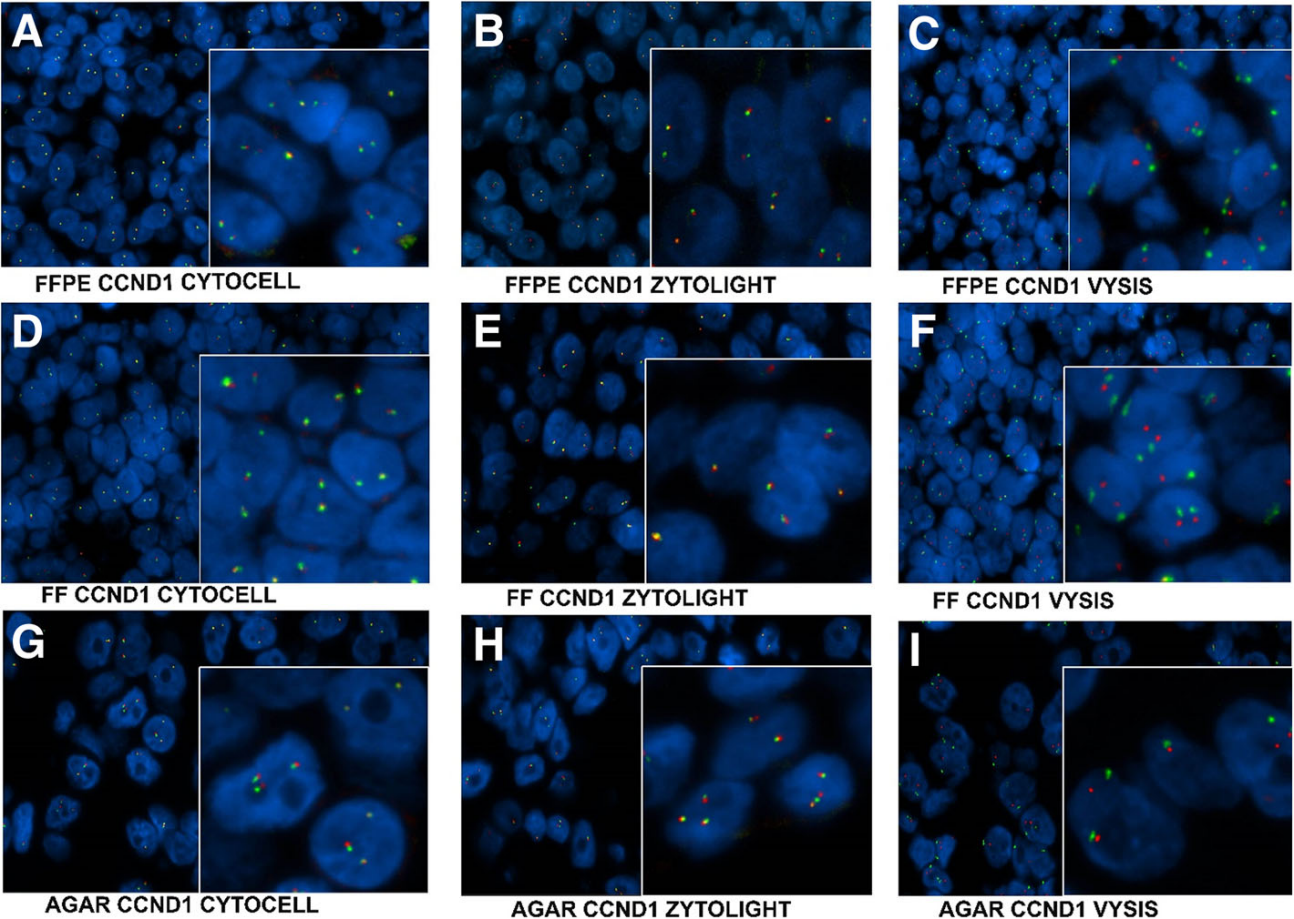
Representative FISH images of FFPE, FF and agar embedded cytological samples with Cytocell, Vysis, and ZytoLight CCND1 FISH probes that were simultaneously processed using the developed standardized procedure. **a** FISH image of FFPE tissue labeled with Cytocell CCND1 Breakapart probe. **b** FISH image of FFPE tissue labeled with ZytoLight SPEC CCND1 Dual Color Break Apart Probe. **c** FISH image of FFPE tissue labeled with CCND1 Vysis LSI Cyclin D1 (11q13) Spectrum Orange/ Vysis CEP 11 Spectrum Green fusion probe. **d** FISH image of FF tissue labeled with Cytocell CCND1 Breakapart probe. **e** FISH image of FF tissue labeled with ZytoLight SPEC CCND1 Dual Color Break Apart Probe. **f** FISH image of FF tissue labeled with CCND1 Vysis LSI Cyclin D1 (11q13) Spectrum Orange/ Vysis CEP 11 Spectrum Green fusion probe. **g** FISH image of cytological agar embedded cells labeled with Cytocell CCND1 Breakapart probe. **h** FISH image of cytological agar embedded cells labeled with ZytoLight SPEC CCND1 Dual Color Break Apart Probe. **i** FISH image of cytological agar embedded cells labeled with CCND1 Vysis LSI Cyclin D1 (11q13) Spectrum Orange/ Vysis CEP 11 Spectrum Green fusion probe

In order to determine the robustness of our optimized pretreatment method on different tissue types with different commercial probes, the FISH analysis was conducted on FFPE, FF and agar embedded Cytological samples simultaneously for one gene. The FFPE, FF and cytological tissue were all healthy specimens. The FFPE and FF samples were tested on tonsil tissue and the cytological samples were tested on agar embedded cells after a lymph node puncture biopsy. As an example for protocol robustness, our new diagnostic protocol was performed with the Cyclin D1 (CCND1) FISH probe from Cytocell, Vysis, and ZytoLight (Fig. 1). The Cytocell and ZytoLight CCND1 probes were break apart probes and the Vysis CCND1 probe was a fusion probe. As expected for healthy tissue, all cases were negative for a gene rearrangement. Our image results show good signal quality with low non-specific background on all tissue types and with Cytocell, Vysis and ZytoLight commercial probes (Fig. 2).

### Protocol evaluation by external quality assessment schemes

Our optimized method achieved 100% concordance for the HER2 FISH NordiQC - Quality Control 2015 evaluation, 100% concordance for genotyping and clerical accuracy for the sarcoma panel containing CHOP (DDIT3), FUS, EWSR1 and MDM2 in the UK NEQAS International Quality Expertise 2016 evaluation. In addition 100% concordance was achieved overall for the sarcoma panel containing SYT (SS18), EWSR1 and FOXO1 in the UK NEQAS International Quality Expertise 2017 evaluation. And lastly 100% concordance was also achieved for the cMYC (MYC) Dutch Foundation for Quality Assessment in Medical Laboratories (SKML) 2017 evaluation.

## Discussion

In this study we present a lean and cost-effective standardized FISH protocol applicable for all probes and tissue types with a high hybridization efficiency. In our cohort of 3881 patient samples, overall 93% of the tested patients samples displayed good quality FISH results leading to a patient diagnosis.

In pathology, the FISH technique is a very useful tool for diagnostic and prognostic purposes [1]. However, clinical testing on patient tissue is challenging due to various variables of tissue processing that may influence the quality of the results. The effectiveness of the FISH technique can be influenced by different factors such as, specimen age, initial tissue handling, specimen fixation, paraffin-embedding and fresh frozen artefacts [10]. The optimization of digestion and post hybridization washing procedures are important for achieving optimal hybridization conditions.

The pretreatment protocol we developed is easy, reproducible, and facilitates FISH on formalin-fixed paraffin-embedded, fresh frozen and cytological patient material with the potential for widespread application in cancer and non-cancer diagnostics and research. In our experience the introduction of an additional formalin fixation step for cytological and fresh frozen samples has significantly increased signal strength and reduced background and therefore the interpretability of these cases.

Previous studies on FISH pretreatment methods described the commonly used pretreatment agent sodium thiocyanate as too harsh when applied to tissue, because tissue sections were found to detach from the slides and were susceptible to mechanical disaggregation [11, 12]. In addition some studies suggested that the optimal concentration, incubation time, and incubation temperatures, of these pretreatment agents must be titrated for the different tissue sections and tissue types [6, 13]. This would suggest the use of multiple protocols for FISH in one laboratory. Our old diagnostic workflow was comprised of various commercial methods from different manufacturers that would be used simultaneously depending on the patient requests. The new diagnostic workflow is lean and uses one in-house developed protocol for all patient samples. Additionally all reagents excluding probes are prepared in-house, making it very cost effective. Leers et al. and Tojo et al. suggested that the heating of cells in an acidic environment with the pretreatment agent, citric acid buffer followed by a proteolytic step improved the fluorescent signals in FFPE tissue significantly [6, 13].

The presented method in this manuscript in combination with the use of citric acid buffer and proteinase K digestion has shown to inflict minimal tissue damage, and to preserve tissue morphology for a correct interpretation of genetic aberrations within the sample. An optimally processed sample is characterized by intact nuclear membranes, non-disturbing autofluorescence and a homogeneous DAPI staining. The majority of the patients in this cohort displayed good signal quality with low non-specific background on all tissue types with different commercial probes. Cases that were classified as uninterpretable were mainly due to increased non-specific background signals, primarily in cases in which additional single signals are of clinical relevance and also generally due to a lack of signal within the nucleus.

Previous FISH methods within our laboratory consisted of multiple kits from various manufacturers that were ideally optimized for a distinct tissue type and probe manufacturer, therefore significantly reducing the amount of slides simultaneously processable per run. The major benefit of our presented protocol is the assurance that all tissue types and different probe manufacturers are processable simultaneously with good quality results.

In terms of appropriate control signals and accuracy of diagnosis, our FISH method has repeatedly confirmed its reliability by means of External Quality Assessment schemes on a national and international level. The optimized pretreatment method has been used to test samples for the NordiQC - Quality Control, UK NEQAS International Quality Expertise and the Dutch Foundation for Quality Assessment in Medical Laboratories (SKML) interlaboratory comparisons studies to check the ability of laboratories to deliver accurate testing results.

Although all variables are well defined in the optimized pretreatment protocol the tissue type will always remain a risk factor. Being a reference center experienced with FISH, specimen are received from various medical centers in the Netherlands and abroad, where each center has their own methods of tissue processing. Nevertheless, those patients included in this cohort performed equally well as our in-house processed samples.

In conclusion the optimized one-fits-all FISH method as presented in this paper is lean, robust, reliable and reproducible for all probes and tissue types facilitating a rapid turnover with a high hybridization efficiency and detection to deliver an accurate diagnosis to patient diagnostics in a cost-effective manner.

## Materials and method

The optimized FISH protocol was used to facilitate routine FISH analysis in a molecular diagnostic setting. FISH analysis was performed on 3881 patient samples included from July 2016 until July 2018 in the University Medical Center Utrecht (The Netherlands) according to the validated pretreatment FISH method for in vitro diagnostic testing (laboratory accreditation ISO15189). Clinical testing on patient material was conducted in compliance to the General Data Protection Regulation (GDPR) as defined by the European Union. Initially, the performance characteristics of 38 commercial probes from Vysis (Abbott Laboratories, Illinois, U.S.A.), Cytocell (Cytocell Ltd., Cambridge, United Kingdom) and ZytoLight (ZytoVision GmbH, Bremerhaven, Germany) were each assessed on 10 normal healthy tissue samples. After validation on healthy tissue slides, probes were tested on diseased tissue material where at least one sample was included that contained the suspected genetic aberration. After validation of the probe, the method is applied in the routine diagnostics setting of the Molecular Pathology department. Tissue samples included in this analysis were formalin-fixed paraffin-embedded (FFPE), fresh frozen (FF), or from cytological specimen.

### Formalin-fixed paraffin-embedded material

FISH analysis was mainly performed on 4-μm FFPE tissue sections mounted on charged slides (Surgipath X-tra Adhesive precleaned Micro slides, Leica Microsystems, Amsterdam, The Netherlands). The tissue slides were incubated at 56 °C for 1–24 h and subsequently deparaffinized in a xylene series and dehydrated in an alcohol series. Formalin-fixed tissue is suitable for clinical diagnostics due to the fact that induced protein-protein and protein-nucleic acid cross-links preserves the tissue efficiently, while retaining morphology relatively intact [12, 14]. However, the macromolecular network introduced by formalin, significantly reduces the access of FISH probes to target DNA [12]. The initial steps in the FISH pretreatment protocol diminishes this formalin induced network.

### Cytological and fresh frozen material

Cytological samples included cellient prepared cells, agar embedded cells, blood smears and cytospin materials [15]. In the case of fresh frozen, cellient and agar blocked material, FISH analysis was performed on 4-μm sections mounted on charged slides. The cellient and agar blocked slides were incubated at 56 °C for 1–24 h and subsequently deparaffinized in a xylene series and dehydrated in an alcohol series. Prior testing has shown an enhanced probe performance when cytological (cellient prepared cells, agar embedded cells, blood smears and cytospin materials) and fresh frozen samples slides were incubated for 16–24 h in a 4% Formaldehyde solution (10% formalin) (Propath bvba, Ronse, Belgium) before proceeding to pretreatment.

### Fluorescence In Situ Hybridization procedure; pretreatment, probe hybridization, post hybridization wash and DAPI counterstaining

The prepared slides were pretreated for 20 min with 0.2 N HCL at room temperature and incubated 20 min at 100 °C in a 10 mM citrate buffer solution pH 6.0. The pretreatment with hydrochloric acid (HCL) aids in solubilizing basic nuclear proteins, improving the accessibility of the DNA. This method extracts the extracellular matrix of proteins which potentially limit the accessibility of the probe to the cells, preventing tissue autofluorescence [12]. Pretreated tissue was digested 10 min in 10 mg/ml digestion buffer pH 7.0 (1 M Tris-HCL, 0.5 M EDTA, 5 M NaCl) and Proteinase K (Sigma- Aldrich, Zwijndrecht, The Netherlands) at 37 °C. Proteinase K digestion is the most crucial step in this protocol in order to obtain technically optimal FISH results [12, 14]. The breaking of peptide bonds by protease digestion directly affects signal quality, allowing access of the FISH probes to the genomic target DNA and reduces autofluorescence generated by intact proteins [12]. Proteinase K digestion was stopped by dehydrating slides in an alcohol series and air-dried. Cytocell and ZytoLight probes were ready to use, Vysis probes were diluted according to manufacturer’s instructions. Throughout the pretreatment validation multiple probe batches were evaluated to determine the variability and reproducibility of the FISH assay. Probes were applied to the tissue slide, cover slipped and sealed with rubber cement. Denaturation was conducted at 78 °C for 5 min and hybridized 12–18 h at 37 °C in a humidified ThermoBrite system (Abbott Molecular, Des Plaines, Illinois, U.S.A.). Post hybridization washing in 2XSSC/0,1% NP-40 pH 7.0 (Boom B.V., Meppel, The Netherlands) to soak off coverslip and stringent wash at preheated temperature of 74 °C in 0.4XSSC/0.3%NP-40 pH 7.0 to remove undesired hybrids of low homology. Subsequently, the slides were rinsed in 2XSSC pH 7.0, then in Phosphate Buffered Saline (PBS) (Lonza, Walkersville, Maryland, U.SA.) and lastly in Milli-Q. Slides were dehydrated in an alcohol series. Air dried slides were counterstained using Vectashield with DAPI hardset mounting medium (Brunschwig Chemie, Amsterdam, The Netherlands) and cover slipped. Slides were stored at − 20 °C if not analyzed immediately.

### Slide imaging and analysis

Before capturing FISH images adjacent hematoxylin and eosin (H&E) stained sections were analyzed for regions containing tumor. These areas were circled using a diamond tipped pen, and the same regions on the FISH slide were examined for molecular DNA aberrations in the nuclei. Following tissue matching, images were captured at 63x magnification with automatic Z-stack and used for analysis on the Leica automatic DM6000 Scanner (Leica Microsystems, Amsterdam, The Netherlands) in combination with CytoVision software (Applied Imaging, Newcastle, United Kingdom). A sufficient number of fields (> 6) per sample were captured and were visualized containing minimally 100 interpretable nuclei. The software analysis was divided into two parts; (1) segmentation, and (2) classification. Segmentation is the identification of nuclei (DAPI stained) by the software and classification involves the identification of nuclear signal patterns (green or red fluorescence probe signals) and their classification in categories (e.g. as non-rearranged or rearranged). Segmentation and classification are error prone, due to the heterogeneity associated with imaging of tissue sections, 3D characteristic of tissue slides in pathology (whereby cells might overlap in images and tissue sectioning artefacts), and Z-stack analysis [16]. The FISH images required manual editing before final analysis, which involved correcting automated classification errors due to inaccurate nuclear detection. Nuclei without signal were manually excluded from the analysis. The remaining nuclei were examined for signal accuracy. Finally, additional nuclei were manually added to the analysis if there were insufficient nuclei identified by the analysis software. Additional nuclei were chosen if the contour could be easily identified. During FISH analysis cells were scored into categories based on probe type (Table 2). The criteria after which a case can then be considered aberrant or not aberrant varies per probe.

**Table 2:** Categories for digital FISH image analysis per probe type

Breakapart probe	Fusion probe	Amplification probe
Normal (2F)	Normal (2R, 2G)	Normal (2R, 2G)
One pair (1F)	One pair (1R, 1G)	Ratio - Not amplified (ratio 1, ≥3R, ≥3G)
Breakapart (1F, 1R, 1G)	Fusion (1(− 2)F)	Ratio - Gain^a^
Single red (1F, 1R)	Single red (2G, 1R)	Ratio - Amplified^a^
Single green (1F, 1G)	Single green (2R, 1G)	Copy number - Not amplified^a^
Extra red (2F, ≥1R)	Extra red (2G, ≥3R)	Copy number - Gain^a^
Extra green (2F, ≥1G)	Extra green (2R, ≥3G)	Copy number - Amplified^a^
Gain (3 - 8F)	Gain (3 – 8R, 3 – 8G)	
Amplification (≥8F)	Amplification (≥8R, ≥8G)	

*F* Fusion signal, *R* Red/Orange signal, *G* Green signal

^a^Criteria varies per probe as described in the legends of Table 1

### Quality of FISH results

During the verification phase of new probes quality requirements were assessed on 10 normal healthy tissue samples prior to implementation. It is required that the adequacy and consistency of signal strength (brightness), lack of background and / or cross-hybridization signals, presence of appropriate control signals, sensitivity, specificity, reproducibility and stability between runs be determined. For a probe to be considered reliable for implementation within the routine diagnostics ≥95% of cases evaluated need to be considered interpretable (acceptable). If this is not achieved the probe was considered unreliable for diagnostic application.

Considering our FISH application within a routine diagnostic laboratory, quality guidelines are established to assure reliable patient results are reported. All diagnostic patients in this cohort were random. Patients samples were not grouped into different quality groups but were considered of sufficient quality or not to allow a reliable patient result to be given. Therefore the required quality aspects (adequacy and consistency of signal strength (brightness), lack of background and / or cross hybridization signals, and additionally the presence of appropriate control signals) were evaluated for each patient after which the results obtained from each diagnostic patient is considered interpretable or uninterpretable. Patient samples with interpretable results were considered for further treatment or diagnosis whereas uninterpretable (unacceptable) results were not. These uninterpretable FISH results were characterized by a poor cellular morphology, loss of /or low fluorescent signal quality, and /or high background fluorescence signals from which no reliable patient diagnosis can be given. In the case of uninterpretable results alternative diagnostic experiments are conducted.

## Data Availability

The datasets generated and analyzed during the current study are not publicly available due to the conflicts with the General Data Protection Regulations but are available from the corresponding author on reasonable request.

## Abbreviations

FF: Fresh frozen
FFPE: Formalin-fixed paraffin-embedded
FISH: Fluorescence In Situ Hybridization
H&E: Hematoxylin and eosin
